# Supplementary material for: Reconciling Mining with the Conservation of Cave Biodiversity: A Quantitative Baseline to Help Establish Conservation Priorities
Source: PLoS One. 2016 Dec 20;11(12):e0168348. doi: 10.1371/journal.pone.0168348 (PMC5173368; doi:10.1371/journal.pone.0168348)
Supplement: S1 Dataset — (ZIP) [file pone.0168348.s002.zip › Taxa/Serra Norte/SN_2013/N4E/Lista N4E-48.pdf]

CAVIDADE N4E-0048

Classe	Ordem	Fam/Outros	Gên/Outros	Espécie	S-U	U-S	Zon.
Arachnida	Acari	Cunaxidae		sp.1		X	E
Arachnida	Acari	Erythraeidae		sp.64	X		E/P
Arachnida	Acari	Ixodidae		jovem	X		P
Arachnida	Acari	Neothyridae	*Diplothyrus*	sp.1		X	P
Arachnida	Amblypygi	Charinidae	*Charinus*	sp.1	1		P
Arachnida	Araneae	Drymusidae		jovem		X	P
Arachnida	Araneae	Filistatidae		jovem	X	X	E
Arachnida	Araneae	Ochyroceratidae	*Ochyrocera*	sp.3		X	P
Arachnida	Araneae	Pholcidae	*Mesabolivar*	sp.1	X	X	E/P
Arachnida	Araneae	Pholcidae	Ninetinae	sp.1	X	X	P
Arachnida	Araneae	Theridiosomatidae	*Plato*	sp.1		X	P
Arachnida	Opiliones	Escadabiidae		sp.1		X	P
Diplopoda	Polydesmida	Pyrgodesmidae		sp.1		X	P
Diplopoda	Spirostreptida	Pseudonannolenidae	*Pseudonannolene*	jovem	2		P
Entognatha	Collembola	Entomobryidae		sp.1	X		E/P
Entognatha	Collembola	Isotomidae		sp.1		X	P
Entognatha	Collembola	Paronellidae		sp.1	X		E/P
Entognatha	Collembola	Paronellidae		sp.4		X	E/P
Entognatha	Diplura	Projapygidae		sp.1	X		P
Insecta	Diptera	Cecidomyiidae	Cecidomyiinae	sp.		X	E
Insecta	Diptera	Chironomidae		sp.		X	E
Insecta	Diptera	Psychodidae	Phlebotominae	sp.	X	X	E/P
Insecta	Diptera	Tipulidae	Tipulinae	sp.	X		E/P
Insecta	Heteroptera	Dipsocoroidea		jovem		X	P
Insecta	Homoptera	Cixiidae		sp.3	X	X	E
Insecta	Homoptera	Cixiidae		sp.5		X	E
Insecta	Hymenoptera	Formicidae	*Pachycondyla*	sp.2		X	P
Insecta	Hymenoptera	Formicidae	*Solenopsis*	sp.1	X		P
Insecta	Hymenoptera	Formicidae	*Trachymyrmex*	sp.1		X	P
Insecta	Lepidoptera	Galacticidae		sp.		X	P
Insecta	Orthoptera	Gryllidae	Eneopterinae	sp.		1	P
Insecta	Orthoptera	Gryllidae	Nemobiinae	sp.		1	
Insecta	Orthoptera	Phalangopsidae	*Paraclodes*	sp.	9		E/P
Insecta	Orthoptera	Phalangopsidae	*Phalangopsis*	sp.	3	5	P
Insecta	Psocoptera			jovem	X		P
Gastropoda	Pulmonata	Systrophiidae	*Happia*	sp.		X	E/P
Symphyla		Scutigerellidae		sp.		X	E/P
Amphibia	Anura	Strabomantidae	*Pristimantis*	cf. *fenestratus*	2		E
Mammalia	Chiroptera	Emballonuridae	*Peropteryx*	*kappleri*		X	E
